# Support for alcohol policies among drinkers in Mongolia, New Zealand, Peru, South Africa, St Kitts and Nevis, Thailand and Vietnam: Data from the International Alcohol Control Study

**DOI:** 10.1111/dar.12647

**Published:** 2017-12-21

**Authors:** Charles D. H. Parry, Mukhethwa Londani, Palam Enkhtuya, Taisia Huckle, Marina Piazza, Gaile Gray‐Phillip, Surasak Chaiyasong, Pham Viet Cuong, Sally Casswell

**Affiliations:** ^1^ Alcohol, Tobacco & Other Drug Research Unit South African Medical Research Council Cape Town and Pretoria South Africa; ^2^ Department of Psychiatry Stellenbosch University Cape Town South Africa; ^3^ National Center for Public Health Ulaanbataar Mongolia; ^4^ SHORE & Whariki Research Centre, College of Health Massey University Auckland New Zealand; ^5^ Facultad de Salud Publica y Administración (FASPA) Universidad Peruana Cayetano Heredia Lima Peru; ^6^ Division of Arts, Sciences and General Studies Clarence Fitzroy Bryant College Basseterre St Kitts and Nevis; ^7^ Social Pharmacy Research Unit, Faculty of Pharmacy Mahasarakham University Maha Sarakham Thailand; ^8^ Health Promotion Policy Research Center International Health Policy Program Thailand; ^9^ Hanoi School of Public Health Hanoi Vietnam

**Keywords:** alcohol, policy, policy analysis, drinking risk, international comparison

## Abstract

**Introduction and Aims:**

A 2010 World Health Assembly resolution called on member states to intensify efforts to address alcohol‐related harm. Progress has been slow. This study aims to determine the magnitude of public support for 12 alcohol policies and whether it differs by country, demographic factors and drinking risk (volume consumed).

**Design and Methods:**

Data are drawn from seven countries participating in the International Alcohol Control Study which used country‐specific sampling methods designed to obtain random, representative samples. The weighted total sample comprised 11 494 drinkers aged 16–65 years.

**Results:**

Drinking risk was substantial (24% ‘increased’ risk and 16% ‘high’ risk) and was particularly high in South Africa. Support varied by alcohol policy, ranging from 12% to 96%, but was above 50% for 79% of the possible country/policy combinations. Across countries, policy support was generally higher for policies addressing drink driving and increasing the alcohol purchase age. There was less support for policies increasing the price of alcohol, especially when funds were not earmarked. Policy support differed by country, and was generally higher in the five middle‐income countries than in New Zealand. It also differed by age, gender, education, quantity/frequency of drinking, risk category and country income level.

**Discussion and Conclusions:**

We found a trend in policy support, generally being highest in the low–middle‐income countries, followed by high–middle‐income countries and then high‐income countries. Support from drinkers for a range of alcohol policies is extensive across all countries and could be used as a catalyst for further policy action.

## Introduction

Alcohol was the ninth leading risk factor for death and disability globally in 2015 1. Generally, the higher the volume of alcohol consumed, the higher the risk of disease or death 2. In addition to the volume of alcohol consumption, negative health outcomes have been found to be dependent on the pattern of drinking, as well as societal and individual vulnerability factors 3.

Since 2010 the World Health Organization has been active in prompting member states to take more action to address harmful use of alcohol. In that year its *Global Strategy to Reduce the Harmful Use of Alcohol* was approved by Member States 4. Within the strategy, policy options and interventions available for national action are grouped into 10 areas: (i) leadership, awareness and commitment; (ii) health services’ response; (iii) community action; (iv) drink‐driving policies and countermeasures; (v) availability of alcohol; (vi) marketing of alcoholic beverages; (vii) pricing policies; (viii) reducing the negative consequences of drinking and alcohol intoxication; (ix) reducing the public health impact of illicit and informally produced alcohol; and (x) monitoring and surveillance 4.

There have been few evaluations of the extent to which individual countries or regions have progressed in implementing policies in line with the *Global Strategy*. From assessments undertaken in Canada, Finland and South Africa it appears that some progress is being made, but despite the growing evidence base regarding what works, there remains much to be done 5, 6. The approval and implementation of public policies around alcohol is influenced by various factors, including the views of a determined bureaucracy 7, the desire by governments to generate revenues, the cost of implementing the policies, research findings, public opinion and industry lobbying 8, 9, 10, 11. Public opinion, while sometimes being a spur to policy reform, can also constrain the policies supported by governments 11. Furthermore, public opinion regarding different alcohol policies at country level has been found to change over time 12, 13, 14.

It has now been well established that support for more restrictive alcohol policies tends to be highest among light or non‐drinkers 8, 10, 11, 12, 14, 15, 16, 17 and when those policies are aimed at high‐risk venues and populations 18. In terms of the effect of demographics, support has been found to greatest among older persons 8, 10, 11, 12, 14, 15, 16, 17, 18, 19 and women 8, 10, 11, 12, 14, 18, 19. However, research findings regarding the effect of education and income on policy support have been mixed 8, 11, 14, 17.

In most other studies looking at public support for alcohol policies the views of drinkers and non‐drinkers have been canvassed 10, 11, 12, 13, 14, 18, 19, 20, 21. The views of non‐drinkers regarding alcohol policy options are likely to be more supportive than those of moderate and heavy drinkers or those who drink more frequently 8, 10, 11, 12, 14, 15, 16, 19. Policymakers would expect the latter to oppose many policy measures and their views might be of particular interest 10.

Generally, support for policies increasing the legal alcohol purchase age and increasing drink‐driving counter measures have received more support than those limiting availability or aimed at increasing the price of alcohol 8, 11, 18, 22. However, few, if any, studies have compared support for different alcohol policy across different countries in any systematic way. The purpose of this study therefore was to assess public support for a range of alcohol control measures covering the following domains (purchase age, physical availability, marketing, drink driving counter measures and price) across samples in seven countries using similar methodology and to determine whether this support varies by country (and country income level), demographic factors and quantity/frequency of drinking and level of drinking risk.

## Methods

### *Design and sampling*

Data for this study come from the multi‐country International Alcohol Control (IAC) Study 23, and this paper focuses on data from two small high‐income countries [New Zealand (2011) and St Kitts and Nevis (2014/16)], three high–middle‐income countries [Peru (2015), South Africa (2014) and Thailand (2012/13)] and two low–middle‐income countries [Mongolia (2013) and Vietnam (2014)]. The IAC countries were largely self‐selected depending on availability of resources and to ensure a spread of countries around the world. The individual countries used different methodologies designed to obtain random, representative samples, such as a multi‐stage stratified cluster random sampling design in Peru, South Africa, St Kitts and Nevis, Mongolia, Thailand and Vietnam and a representative sample of published and unpublished residential landline numbers in New Zealand. For New Zealand and St Kitts and Nevis the focus was the whole country, whereas for other countries the focus was on sub‐national samples: two districts in Ulaanbaatar in Mongolia, Los Olivos district in the city of Lima in Peru, Tshwane metro in South Africa, nine provinces in Thailand (including Bangkok) and three provinces in Vietnam. In this sub‐study, and in line with the broader IAC study 23, eligible participants had to have consumed alcohol in the past 6 months and be 16 to 65 years old. Levels of non‐drinking ranged between 20.5% in New Zealand to 70.3% in Thailand 3, but fell between 45% and 62% for the other five countries. The IAC study was designed to be a longitudinal study of drinkers with the focus of assessing the impact of policy changes on the behaviour of drinkers within and across countries over time 23. This focus on drinkers was predicated on the overarching goal of the study to reduce the harms associated with the behaviour of drinkers to themselves and others through guiding upstream policy‐level interventions that would impact on such behaviour. The target sample size of 2000 per country was determined by the IAC study 23.

### *Measures*

Countries adapted the English IAC questionnaire and translated and back‐translated it into local languages (Mongolia, Peru, South Africa, Thailand and Vietnam) and piloted it before use. It comprised various domains, including demographic factors (e.g. age, gender and education), alcohol consumption and support for 12 alcohol policies. The latter was obtained on a 5‐point Likert scale ranging from ‘strongly support’ (1) to ‘strongly oppose’ (5) with ‘do not know’ and ‘refuse’ options. Strongly support (1) and support (2) were combined to indicate support, and neutral (3), oppose (4) and strongly oppose (5) were combined to indicate lack of support. Persons who answered ‘do not know’ or refused to answer the question were excluded for that policy. For Thailand, three policies (10–12, see Table 2) and one for Vietnam (policy 8) were not included. We created a summary index which is the sum of the scores on the 12 individual policy support variables. This total policy support variable (‘*Policysum’*) yields for each study participant a score from 12 (strong support) to 60 (strong opposition) which is a crude measure of their support for the various policy variables as a whole.

The IAC questionnaire utilises a within‐location beverage‐specific framework and allows for countries to adapt the consumption measurement framework to their context in terms of specific drinking locations. The framework asks for the frequency of drinking in all locations in which drinking occurs and then typical occasion quantity in each location. The IAC consumption framework asks beverage‐specific questions for each location at which participants drink.

Using the data on frequency of drinking in different locations over the past 6 months and quantity consumed on a typical occasion in each location, new variables were derived at the analysis stage. ‘Frequency of drinking’ is the sum of all drinking occasions at all locations over the past 6 months. The ‘typical occasion quantity’ is the weighted average of all the typical occasion quantities at each location, taking into account how often the person drank at that location. In this way, a location that a person only drank at once a year had minimal influence compared with a location that a person drank at daily. It was categorised into ^‘^low’ (>4 drinks), ‘medium’ (4–6 drinks), ‘medium‐high’ (6 to ≤ 8 drinks) and ‘high’ (>8 drinks). Respondents reported their consumption of different beverages specific to their country in their own terms and interviewers coded these by using common containers and glass sizes in which alcohol is commonly served and sold in that country. We therefore had interviewers code what study participants said they consumed and we then afterwards recorded this into the number of drinks of 15 mL (12 g). ‘Risk category’ (drinking risk) was categorised into three levels: ‘low’ (up to four drinks on an occasion or four to six drinks on an occasion less than once per week), ‘increased’ (four to six drinks on an occasion at least once per week or more than six drinks on an occasion less than once per week) and ‘higher’ (more than six drinks on an occasion at least once per week). The risk categories were therefore derived based on the original location‐based occasion (not the overall summary typical occasion quantity). Where participants did not provide complete data on quantity and frequency of drinking in all locations their data were omitted.

### *Procedures*

Some of the countries used telephone surveys (New Zealand) whereas others interviewed participants in their homes (Mongolia, Peru, South Africa, St Kitts and Nevis, Thailand and Vietnam). Computer‐assisted surveys were used in all countries due to the complexity of the survey with numerous skip patterns. This involved using android tablets in Mongolia, Peru, South Africa, St Kitts and Nevis, Thailand and Vietnam, and an in‐house computer‐assisted telephone interviewing system in New Zealand. Once a household was contacted, a screening interview established eligibility for participation in the study (drinking in the last 6 months and age 16–65 years). Eligible individuals were enumerated, and one respondent was selected at random by the computer/tablet. Informed consent was then sought from the person identified before proceeding. Interviews took on an average about 30 minutes to complete. In many of the countries (e.g. New Zealand, South Africa and Thailand) participants received a small gift (e.g. a modest shopping or a cellular telephone recharge voucher or a Polo shirt) to acknowledge their participation. The studies were approved by their local ethics committees. Response rates varied from 60% in New Zealand and St Kitts and Nevis to 99% in Vietnam.

### *Survey design and analysis*

It is important to account for the sampling design during analysis to make sure that the standard errors are not underestimated (i.e. where modelling or statistical testing is being undertaken). This was not able to be done simply due to each of the countries in our study having different sampling designs, ranging from a simple random to a stratified multistage sample. The process used to adjust for cross‐country sampling design was based on Kaminska and Lynn 2017 24 which treats the individual countries as the top level strata. For countries (New Zealand, St Kitts and Nevis, South Africa, Australia and Thailand) which had already been stratified at the first stage, these strata became first stage strata of the combined survey. Countries (Mongolia, England, Scotland, Vietnam) that do not have first stage stratification were treated as a single stratum. Checks were made so that stratum identifications were still unique after combining countries. Primary sampling units remained the same, again making sure that they were still unique after combining all countries. Weights were used where available and assumed to have a weight of one where they were not. Finite population correction was not used as this was incomplete for the majority of countries.

A statistical process was used to deal with outliers whereby the right‐skewed distributions of consumption‐related variables were transformed to normalise them. The transforming function was logarithmic (for typical occasion quantity) and power function (for frequency of drinking). The transformed series was then centred and scaled by subtracting the mean and dividing by the standard deviation and the 99th percentile of respondents were then removed. Using Stata 25 multiple logistic regression analyses were performed to assess associations between predictors and each of the 12 policy options separately. Categorical predictors for each policy option included country, gender, age, education, drinking frequency, drinking quantity and drinking risk. Responses on policy variables of ‘support’ and ‘strongly support’ were recoded as ‘1’ and ‘neither’, ‘oppose’ and ‘strongly oppose’ were recoded as ‘0’. Adjusted odds ratios were used to control for the potential effect of additional variables. Participants who answered ‘Do not know’ to a specific policy option were excluded from that logistic regression model. Participants with missing data were excluded from all logistic regressions. *P* values less than 0.05 were considered statistically significant. A second (post‐hoc) multiple regression was undertaken to assess the association between the policy support variables and a newly constituted variable, country‐income level [low–middle‐income, high–middle‐income (reference), high income] adjusted for age, gender education and drinking risk.

## Results

### *Sample characteristics*

The weighted sample of drinkers included 11 494 respondents aged 16 to 65 years (Table 1). The most common age categories were 25–34, 35–44 and 45–54 years. In all countries there were more male than female drinkers, except in New Zealand and Peru. In three countries (Peru, Thailand and Vietnam), the highest proportion of respondents were reported having low education. For four of the seven countries (New Zealand, South Africa, St Kitts and Nevis and Vietnam), the most commonly reported category of drinking was once a week or more. For other countries, it was less often than this. For all countries, except for South Africa, the greatest proportion of drinkers fell into the lowest quantity of drinking category. Similarly, for all countries barring South Africa, the greatest proportion of respondents (46% to 74%) reported drinking fewer than four drinks on a typical drinking occasion, whereas in South Africa only 22% of respondents reported drinking at this level and 54% reported drinking more than eight. In terms of risk associated with drinking, for all countries except South Africa, the largest proportion of respondents fell into the lowest category of risk.

**Table 1:** Demographic and drinking characteristics of study participants (drinkers only) in the seven countries (column %, adding up to 100%) (income level)

	Mongolia (L–M)	New Zealand^a^ (H)	Peru^a^ (H–M)	South Africa^a^ (H–M)	St Kitts and Nevis (H)	Thailand^a^ (H–M)	Vietnam (L–M)
**Demographic variables**							
*Age, years*							
16–17	2.1	4.3	3.0	3.6	2.3	2.9	0.2
18–19	1.4	6.5	5.5	6.1	5.2	2.6	0.3
20–24	9.8	6.2	11.8	17.7	19.2	6.1	2.9
25–34	26.7	15.6	23.7	28.2	27.4	15.6	14.8
35–44	22.6	25.1	20.0	18.4	20.1	25.4	28.0
45–54	22.3	21.4	18.4	14.6	14.6	29.5	29.8
55–65	15.1	21.0	17.5	11.3	11.2	17.9	24.0
*Gender*							
Male	53.7	41.8	45.6	64.7	66.1	57.2	90.8
Female	46.3	58.2	54.4	35.3	33.9	42.8	9.2
*Education* b							
Low	10.3	9.8	56.9	30.1	39.4	57.5	64.6
Mid	31.6	45.1	19.9	57.2	50.9	21.1	15.2
High	58.2	45.1	23.2	12.7	9.7	21.4	20.3
**Drinking characteristics**							
*Drinking frequency* c							
Low	40.8	8.9	64.4	32.6	15.1	43.1	13.3
Med	43.2	16.5	30.8	18.3	16.7	15.9	27.4
High	16.0	74.5	4.8	49.1	68.2	41.0	59.3
*Drinking quantity* d							
Low	48.1	72.2	74.4	22.2	53.2	66.5	60.6
Med	28.1	12.5	10.6	10.3	20.4	15.9	18.7
Med–High	9.3	6.5	7.0	14.0	8.8	7.4	7.7
High	14.5	8.7	8.0	53.6	17.6	10.3	13.1
*Risk category* e							
Low	54.6	61.4	77.6	24.7	45.7	64.3	60.3
Increased	37.6	21.8	21.4	38.7	24.9	21.4	20.4
Higher	7.8	16.8	1.0	36.7	29.4	14.3	19.3
**Total** f	862	2001	1886	1007	1343	2377	2018

a Weighted data.

b Low: up to 10 years, Mid: 11–13 years, High: more than 13 years.

c Low: up to once/month, Med: more than once/month but less than once/week, High: once a week or more (among drinkers).

d On typical occasion in past 6 months (among drinkers). Low: >4 drinks, Med: 4–6 drinks, Med–High: 6 to ≤ 8 drinks, High: >8 drinks.

e Low risk: up to 4 drinks on an occasion or 4–6 drinks on an occasion less than once/week, increased: 4–6 drinks on an occasion at least once/week or 6+ drinks on an occasion less than once/week, Higher: 6+ drinks on an occasion at least once/week.

f Total drinkers in (unweighted) sample. For some variables there was missing data for some countries. Overall *N* (unweighted samples) was 11 494 over 7 countries. The total for the weighted sample was 11 526. H, high; L, low; M, middle.

### *Policy support*

**Figure 1:**
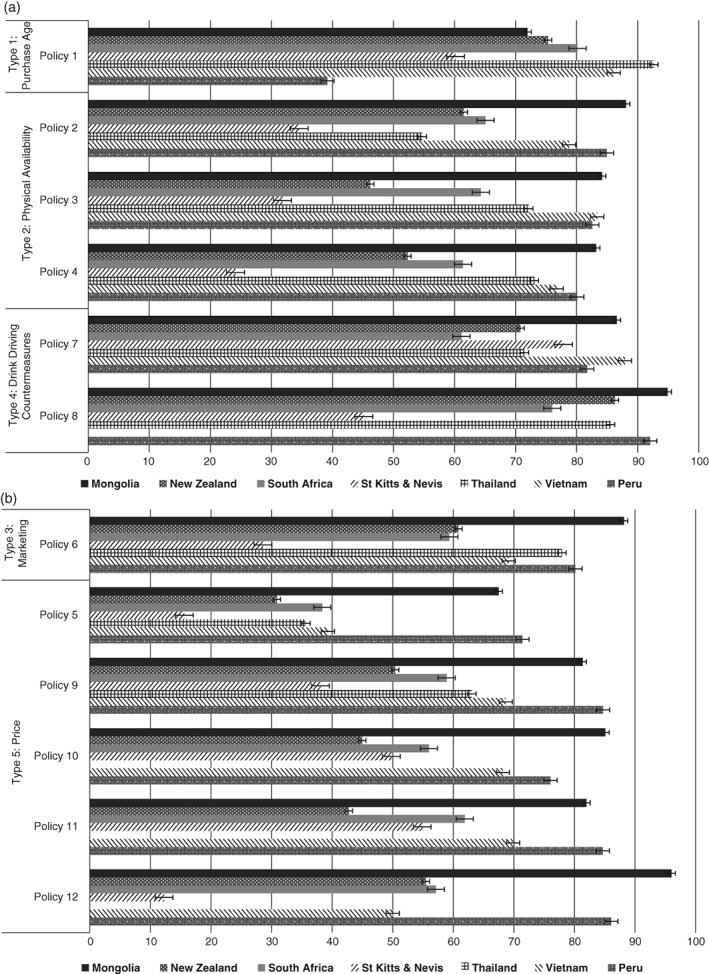
(a) Support for alcohol policies dealing with purchase age, physical availability and drink driving (%) and 95% error bars [Policy 1: a purchase age of 20 years (18 years for South Africa); Policy 2: restrictions of number of outlets in your community; Policy 3: earlier closing times for bars and nightclubs; Policy 4: earlier closing times for buying alcohol from bottle stores and supermarkets; Policy 7: lowering the blood/breath alcohol limit for drink driving; Policy 8: more random breath testing]. (b) Support for alcohol policies dealing with marketing and price (%) [Policy 5: an increase in the price of alcohol; Policy 6: restrictions on alcohol marketing; Policy 9: an increase in alcohol taxes to pay for alcohol treatment; Policy 10: an increase in alcohol taxes to lower other taxes; Policy 11: an increase in alcohol taxes to pay for any government purpose; Policy 12: taxing drinkers to pay for the cost of alcohol‐related harm to society].

Support varied by alcohol policy, ranging from 12% to 96%, with support above 50% for 63 (79%) of the possible 80 country/policy combinations (Figure 1a,b). Support for the various policies was generally highest in Mongolia (67–96%) and Peru (71–92%, excluding the 39% support for increasing the purchase age) and lowest in St Kitts and Nevis (12–78%). Across countries, support was higher for policies addressing drink driving and increasing the purchase age (except for Peru). Support for increasing price was in general lower than for the other policy areas (16–71%), and compared to the pricing policies for which more detail was given. The created variable policysum had scores ranging from 12 to 60 and a mean of 26.6 and standard deviation of 8.18. The mode for ‘policysum’ was 24 with 655 participants having this score.

### *Multivariate findings*

The level of support for policies in New Zealand was used as the standard category for comparison. Table 2 presents 75 country/policy comparisons. Of these, 68 comparisons revealed significant differences on policies as compared with New Zealand. There were 52 instances (77%) where policy support was greater than occurred in New Zealand and 15 (22%) where it was less, with the latter mainly occurring as a result of policy support being significantly less for various (10) policies in St Kitts and Nevis. In most cases, the size of the adjusted odds ratios indicated that the differences in terms of policy support between the various countries and New Zealand were substantial. This was most noticeable for Mongolia and Vietnam. There were seven comparisons where there were no significant differences in policy support (three with South Africa, two with Thailand and one each with Mongolia and Vietnam).

**Table 2:** Multiple logistic regression with policy support variables (as DVs) and country, demographic and drinking variables (as IVs)

	Policysum			Policy 1			Policy 2			Policy 3			Policy 4		
	AOR	95% CI	*P* value	AOR	95% CI	*P* value	AOR	95% CI	*P* value	AOR	95% CI	*P* value	AOR	95% CI	*P* value
*Country*															
New Zealand^a^	(Ref)			(Ref)	—	—	(Ref)	—		(Ref)	—		(Ref)	—	
Mongolia	5.62	4.42, 7.15	<0.001	0.69	0.56, 0.85	0.001	4.34	3.38, 5.57	<0.001	8.05	6.32, 10.26	<0.001	4.87	3.87, 6.14	<0.001
Peru^a^	1.90	1.48, 2.45	<0.001	0.15	0.13, 0.19	<0.001	2.95	2.36, 3.69	<0.001	5.78	4.57, 7.30	<0.001	3.25	2.62, 4.02	<0.001
South Africa^a^	1.89	1.39, 2.57	<0.001	1.34	0.95, 1.89	0.096	1.44	0.95, 2.18	0.085	3.51	2.51, 4.89	<0.001	2.03	1.55, 2.65	<0.001
St Kitts and Nevis	0.54	0.42, 0.69	<0.001	0.47	0.39, 0.56	<0.001	0.42	0.35, 0.51	<0.001	0.80	0.66, 0.97	0.021	0.39	0.32, 0.47	<0.001
Thailand^a^	14.25	11.11, 18.28	<0.001	3.68	3.08, 4.39	<0.001	0.67	0.56, 0.81	<0.001	3.02	2.23, 4.09	<0.001	2.31	1.86, 2.86	<0.001
Vietnam	4.61	2.10, 10.12	<0.001	1.68	1.25, 2.26	0.001	2.48	1.57, 3.91	<0.001	6.41	3.52, 11.67	<0.001	3.12	1.84, 5.28	<0.001
*Gender*															
Male	(Ref)			(Ref)	—	—	(Ref)	—		(Ref)	—		(Ref)	—	
Female	1.34	1.18, 1.51	<0.001	1.17	1.05, 1.31	0.006	1.31	1.18, 1.46	<0.001	1.47	1.31, 1.64	<0.001	1.42	1.26, 1.59	<0.001
*Age, years*															
16–19	(Ref)			(Ref)	—	—	(Ref)	—		(Ref)	—		(Ref)	—	
20–24	1.27	0.96, 1.67	0.091	3.23	2.51, 4.16	<0.001	1.25	0.99, 1.59	0.064	1.12	0.87, 1.44	0.374	1.16	0.90, 1.49	0.251
25–34	1.68	1.31, 2.17	<0.001	4.41	3.50, 5.55	<0.001	1.65	1.34, 2.03	<0.001	1.82	1.43, 2.31	<0.001	1.41	1.08, 1.83	0.012
35–44	1.88	1.44, 2.45	<0.001	4.13	3.32, 5.13	<0.001	1.81	1.49, 2.20	<0.001	2.40	1.88, 3.06	<0.001	2.02	1.58, 2.58	<0.001
45–54	2.47	1.92, 3.18	<0.001	3.86	3.11, 4.79	<0.001	2.41	1.98, 2.94	<0.001	3.78	2.96, 4.82	<0.001	2.54	1.94, 3.32	<0.001
55–65	2.95	2.25, 3.87	<0.001	3.94	3.11, 5.00	<0.001	2.50	1.98, 3.16	<0.001	4.21	3.28, 5.39	<0.001	2.72	2.10, 3.54	<0.001
*Education* b															
Low	(Ref)			(Ref)	—	—	(Ref)	—		(Ref)	—		(Ref)	—	
Middle	0.88	0.73, 1.05	<0.001	0.89	0.76, 1.04	0.142	1.04	0.86, 1.26	0.680	0.89	0.72, 1.10	0.263	0.91	0.73, 1.13	0.381
High	1.03	0.80, 1.33	<0.001	0.88	0.73, 1.07	0.199	1.10	0.88, 1.38	0.386	0.88	0.66, 1.17	0.369	0.92	0.70, 1.21	0.564
*Drinking frequency* c															
Low	(Ref)			(Ref)	—	—	(Ref)	—		(Ref)	—		(Ref)	—	
Middle	0.71	0.63, 0.81	<0.001	0.98	0.86, 1.13	0.819	0.87	0.72, 1.03	0.111	0.86	0.72, 1.01	0.066	0.88	0.75, 1.04	0.127
High	0.67	0.53, 0.83	<0.001	0.85	0.72, 1.01	0.061	0.66	0.55, 0.79	<0.001	0.82	0.70, 0.97	0.020	0.73	0.63, 0.85	<0.001
*Drinking quatity* d															
Low	(Ref)			(Ref)	—	—	(Ref)	—		(Ref)	—		(Ref)	—	
Middle	0.91	0.75, 1.10	0.311	1.03	0.87, 1.22	0.748	0.77	0.65, 0.92	0.004	0.66	0.55, 0.79	<0.001	0.77	0.64, 0.93	0.006
Mid–High	0.92	0.71, 1.18	0.496	0.97	0.75, 1.25	0.812	0.82	0.65, 1.04	0.109	0.62	0.50, 0.77	<0.001	0.71	0.56, 0.89	0.002
High	0.91	0.68, 1.22	0.522	1.12	0.88, 1.43	0.343	0.83	0.63, 1.09	0.169	0.65	0.51, 0.82	<0.001	0.83	0.64, 1.09	0.176
*Risk drinking* e															
Low	(Ref)			(Ref)	—	—	(Ref)	—		(Ref)	—		(Ref)	—	
Increased	0.82	0.67, 1.01	0.058	0.87	0.74, 1.02	0.075	0.96	0.82, 1.12	0.563	1.02	0.86, 1.21	0.816	0.87	0.75, 1.01	0.062
Higher	0.62	0.43, 0.89	0.010	0.75	0.59, 0.96	0.022	0.92	0.69, 1.23	0.557	0.98	0.75, 1.30	0.906	0.82	0.64, 1.05	0.119

	Policy 5			Policy 6			Policy 7			Policy 8		
	AOR	95% CI	*P* value	AOR	95% CI	*P* value	AOR	95% CI	*P* value	AOR	95% CI	*P* value
*Country*												
New Zealand^a^	(Ref)	—	—	(Ref)	—		(Ref)	—		(Ref)	—	
Mongolia	4.59	3.54, 5.95	<0.001	5.11	3.94, 6.63	<0.001	2.95	2.29, 3.81	<0.001	2.79	1.92, 4.05	<0.001
Peru^a^	4.78	3.78, 6.05	<0.001	2.62	2.12, 3.25	<0.001	1.58	1.27, 1.97	<0.001	1.39	1.06, 1.82	0.018
South Africa^a^	2.01	1.56, 2.58	<0.001	1.31	1.05, 1.63	0.016	0.72	0.55, 0.95	0.018	0.57	0.41, 0.80	0.001
St Kitts and Nevis	0.57	0.47, 0.71	<0.001	0.35	0.29, 0.42	<0.001	1.86	1.53, 2.27	<0.001	0.15	0.12, 0.18	<0.001
Thailand^a^	1.15	0.94, 1.41	0.183	2.38	1.93, 2.93	<0.001	0.91	0.71, 1.17	0.458	0.82	0.61, 1.08	0.155
Vietnam	1.59	0.75, 3.37	0.225	1.64	0.95, 2.82	<0.001	3.02	1.85, 4.93	<0.001	—	—	—
*Gender*												
Male	(ref)	—	—	(Ref)	—		(Ref)	—		(Ref)	—	
Female	1.16	1.05, 1.29	0.004	1.32	1.17, 1.49	<0.001	1.47	1.32, 1.64	<0.001	1.52	1.28, 1.81	<0.001
*Age, years*												
16–19	(ref)	—	—	(Ref)	—		(Ref)	—		(Ref)	—	
20–24	1.16	0.87, 1.56	0.321	1.29	1.02, 1.62	0.031	0.94	0.73, 1.20	0.608	1.18	0.91, 1.53	0.204
25–34	1.46	1.18, 1.81	<0.001	1.43	1.16, 1.76	0.001	1.13	0.92, 1.38	0.233	1.60	1.26, 2.03	<0.001
35–44	1.77	1.39, 2.25	<0.001	1.84	1.51, 2.23	<0.001	1.32	1.05, 1.65	0.016	2.20	1.71, 2.82	<0.001
45–54	1.96	1.54, 2.49	<0.001	2.22	1.81, 2.74	<0.001	1.49	1.20, 1.84	<0.001	1.93	1.50, 2.49	<0.001
55–65	2.39	1.89, 3.03	<0.001	2.57	2.11, 3.13	<0.001	1.73	1.42, 2.11	<0.001	2.07	1.57, 2.71	<0.001
*Education* b												
Low	(Ref)	—	—	(Ref)	—		(Ref)	—		(Ref)	—	
Middle	1.01	0.85, 1.19	0.911	1.09	0.91, 1.30	0.339	0.82	0.67, 1.01	0.066	0.86	0.73, 1.01	0.064
High	1.31	1.08, 1.58	0.005	1.25	0.97, 1.60	0.079	0.81	0.63, 1.04	0.095	0.91	0.75, 1.09	0.295
*Drinking frequency* c												
Low	(Ref)	—	—	(Ref)	—		(Ref)	—		(Ref)	—	
Middle	0.67	0.58, 0.78	<0.001	0.89	0.76, 1.04	0.137	0.85	0.73, 0.99	0.036	0.78	0.65, 0.94	0.008
High	0.66	0.51, 0.85	0.001	0.86	0.73, 1.02	0.074	0.85	0.73, 0.97	0.019	0.67	0.55, 0.83	<0.001
*Drinking quantity* d												
Low	(Ref)	—	—	(Ref)	—		(Ref)	—		(Ref)	—	
Middle	0.87	0.73, 1.05	0.152	0.84	0.69, 1.01	0.067	1.00	0.85, 1.17	0.967	0.79	0.63, 0.99	0.036
Mid–High	1.13	0.89, 1.42	0.334	0.88	0.70, 1.10	0.255	1.08	0.85, 1.36	0.543	0.72	0.52, 1.00	0.048
High	0.96	0.77, 1.20	0.715	0.89	0.71, 1.10	0.283	1.09	0.86, 1.40	0.469	0.90	0.65, 1.25	0.535
*Risk drinking* e												
Low	(Ref)	—	—	(Ref)	—		(Ref)	—		(Ref)	—	
Increased	0.65	0.56, 0.77	<0.001	0.92	0.77, 1.09	0.330	0.83	0.71, 0.98	0.025	1.10	0.87, 1.39	0.410
Higher	0.51	0.38, 0.68	<0.001	0.75	0.56, 1.01	0.054	0.78	0.60, 1.01	0.059	0.88	0.62, 1.24	0.470

	Policy 9			Policy 10			Policy 11			Policy 12		
	AOR	95% CI	*P* value	AOR	95% CI	*P* value	AOR	95% CI	*P* value	AOR	95% CI	*P* value
*Country*												
New Zealand^a^	(Ref)	—	—	(Ref)	—		(Ref)	—		(Ref)	—	
Mongolia	4.16	3.30, 5.25	<0.001	6.05	4.63, 7.90	<0.001	5.71	4.40, 7.40	<0.001	18.47	12.44, 27.43	<0.001
Peru^a^	4.61	3.73, 5.70	<0.001	3.19	2.52, 4.04	<0.001	6.23	4.92, 7.88	<0.001	4.14	3.33, 5.15	<0.001
South Africa^a^	1.66	1.26, 2.17	<0.001	1.50	1.17, 1.91	0.001	2.55	1.96, 3.33	<0.001	1.35	0.94, 1.94	0.109
St Kitts and Nevis	0.73	0.62, 0.87	<0.001	1.32	1.11, 1.56	0.002	1.89	1.59, 2.24	<0.001	0.13	0.10, 0.16	<0.001
Thailand^a^	1.55	1.27, 1.91	<0.001	—	—	—	—	—	—	—	—	—
Vietnam	2.17	1.13, 4.14	0.019	2.82	1.43, 5.56	0.003	3.47	1.60, 7.52	0.002	0.86	0.42, 1.77	0.681
*Gender*												
Male	(Ref)	—	—	(Ref)	—		(Ref)	—		(Ref)	—	
Female	1.08	0.96, 1.20	0.191	1.23	1.07, 1.40	0.003	1.21	1.07, 1.37	0.002	1.24	1.08, 1.43	0.002
*Age, years*												
16–19	(Ref)	—	—	(Ref)	—		(Ref)	—		(Ref)	—	
20–24	1.04	0.87, 1.48	0.335	1.19	0.91, 1.56	0.198	1.22	0.94, 1.59	0.142	1.42	1.06, 1.91	0.020
25–34	1.38	1.09, 1.76	0.008	1.22	0.96, 1.56	0.105	1.29	0.99, 1.69	0.061	1.32	0.99, 1.77	0.062
35–44	1.58	1.26, 1.98	<0.001	1.22	0.96, 1.55	0.105	1.29	1.01, 1.64	0.045	1.37	1.06, 1.76	0.017
45–54	1.65	1.33, 2.05	<0.001	1.33	1.04, 1.70	0.025	1.42	1.10, 1.83	0.008	1.61	1.23, 2.13	0.001
55–65	1.73	1.36, 2.21	<0.001	1.19	0.93, 1.52	0.169	1.35	1.03, 1.77	0.030	1.60	1.22, 2.11	0.001
*Education* b												
Low	(Ref)	—	—	(Ref)	—		(Ref)	—		(Ref)	—	
Middle	1.01	0.89, 1.15	0.869	1.04	0.90, 1.19	0.630	1.05	0.91, 1.21	0.515	0.96	0.85, 1.08	0.457
High	1.07	0.92, 1.24	0.398	1.14	0.97, 1.34	0.112	1.12	0.95, 1.32	0.168	1.17	1.02, 1.35	0.029
*Drinking frequency* c												
Low	(Ref)	—	—	(Ref)	—		(Ref)	—		(Ref)	—	
Middle	0.80	0.71, 0.89	<0.001	0.91	0.78, 1.05	0.197	0.85	0.74, 0.98	0.024	0.89	0.73, 1.10	0.295
High	0.76	0.64, 0.91	0.002	0.76	0.59, 0.96	0.023	0.75	0.59, 0.95	0.018	0.80	0.64, 0.99	0.043
*Drinking quantity* d												
Low	(Ref)	—	—	(Ref)	—		(Ref)	—		(Ref)	—	
Middle	0.87	0.73, 1.03	0.097	1.06	0.84, 1.33	0.636	0.83	0.67, 1.03	0.086	0.92	0.71, 1.19	0.519
Mid–High	0.86	0.69, 1.07	0.180	1.06	0.82, 1.37	0.679	0.81	0.64, 1.04	0.094	0.87	0.67, 1.14	0.309
High	0.91	0.70, 1.19	0.482	1.30	0.94, 1.80	0.116	0.83	0.64, 1.08	0.163	0.92	0.65, 1.29	0.620
*Risk drinking* e												
Low	(Ref)	—	—	(Ref)	—		(Ref)	—		(Ref)	—	
Increased	0.92	0.76, 1.11	0.378	0.90	0.73, 1.12	0.343	0.96	0.78, 1.19	0.726	0.88	0.71, 1.08	0.219
Higher	0.73	0.52, 1.01	0.058	0.81	0.58, 1.14	0.225	0.82	0.57, 1.16	0.263	0.66	0.47, 0.92	0.013

a Weighted data.

b Low: up to 10 years, Mid: 11–13 years, High: more than 13 years.

c Low: up to once/month, Med: more than once/month but less than once/week, High: once a week or more (among drinkers).

d On typical occasion in past 6 months (among drinkers). Low: >4 drinks, Med: 4–6 drinks, Med–High: 6 to ≤ 8 drinks, High: >8 drinks.

e Low risk: up to 4 drinks on an occasion or 4–6 drinks on an occasion less than once/week, increased: 4–6 drinks on an occasion at least once/week or 6+ drinks on an occasion less than once/week, Higher: 6+ drinks on an occasion at least once/week.

AOR, adjusted odds ratio; CI, confidence interval; DV, dependent variable; IV, independent variable.

Support for alcohol policies was higher in five of the six other countries (all middle income) as compared to New Zealand for the following policies: earlier closing times for bars and nightclubs, earlier closing times for buying alcohol from bottle stores and supermarkets, restricting marketing, increasing the price of alcohol, increasing alcohol taxes to pay for alcohol treatment, increasing alcohol taxes to lower other taxes, increasing alcohol taxes to pay for any government purpose (seven of the 12 policies) and also on the ‘policysum’ variable.

Support was 16–52% higher for the other 11 policies among females than males with the exception of increasing alcohol taxes to pay for alcohol treatment (Table 2). In terms of age, participants in age categories older than 20–24 years were generally more supportive of the various alcohol policies than participants in the youngest (16–19 years) age category. There were some exceptions where the adjusted odds ratios, though above 1.0, were not statistically significant. With regard to educational level attained, participants in the highest education levels were significantly more supportive of increasing the price of alcohol in general and specifically increasing price and taxing drinkers to pay for the cost of alcohol‐related harm to society.

Support for various policies was associated with participants’ quantity and frequency of drinking and category of drinking risk. Persons who drank more frequently (or in some cases only those who drank the most frequently) were less likely to support various policies (policies 2–5, 7–12) as compared to participants in the lowest category of frequency of drinking. For Policy 1 (raising the alcohol purchase age) and Policy 6 (restricting marketing) levels of support did not differ by frequency of drinking. With regard to drinking quantity, while in the same direction, fewer significant differences were found. Support for restricting the number of outlets in the community was found to be less for persons who typically drank four to six drinks per occasion (mid‐level drinkers) as compared to persons typically drinking fewer than four drinks, but no differences were found on these items for persons typically drinking more. Persons drinking four or more drinks per occasion were less supportive of earlier closing times for bars and nightclubs and mid‐level and mid‐high (six to less than eight drinks) level drinkers were less likely to be supportive of earlier closing times for buying alcohol from bottle stores and supermarkets and having more random breath testing of drivers. With regard to drinking risk, for some of the policies (policies 1, 5, 7, 12), the higher (or in some cases the highest) level of risk categories were significantly less supportive of these policies than persons who were in the lowest category of risk.

For 10 policies, support was significantly lower (*P* < 0.05) in high‐income countries compared to high–middle‐income countries, and in five instances was significantly higher (*P* < 0.05) in low–middle‐income countries compared to high–middle‐income countries after adjusting for age, gender, education and drinking risk (Table 3). For the constructed variable ‘policysum’, compared to high–middle‐income countries, policy support was significantly lower in high‐income countries, but no difference was found with regard to low–middle‐income countries. With regard to restricting outlets, earlier closing times for bars and nightclubs and more random breath testing, support was higher in low–middle‐income countries and lower in high‐income countries as compared to high–middle‐income countries, after adjusting for age, gender, education and drinking risk (Table 3). There were two policies (increasing alcohol purchase age and lowering blood/breath alcohol limits for drivers) where low–middle‐income countries differed from high–middle‐income countries and no difference was found between high‐income and high–middle‐income countries. In these situations policy support was higher in the low–middle‐income countries as compared to the high–middle‐income countries. There were seven instances where policy support was found to be lower in high‐income countries as compared to high–middle‐income countries and no differences were noted for low‐middle and high–middle‐income countries (policies 4–6, 9–12).

**Table 3:** Multiple logistic regression with policy support variables (DVs) and country‐income level as (IV), adjusted for age, gender, education and drinking risk^a^

	Income level	AOR	95% CI	*P* value
Policysum: sum of scores on the 12 policy variables	High	0.16	0.13–0.19	<0.001
	High–middle	(Ref)	—	—
	Low–middle	0.93	0.58–1.49	0.752
Policy 1: a purchase age of 20 years (18 years for South Africa)	High	0.99	0.78–1.26	0.910
	High–middle	(Ref)	—	—
	Low–middle	1.67	1.18–2.35	0.004
Policy 2: restrictions of number of outlets in your community	High	0.52	0.42–0.65	<0.001
	High–middle	(Ref)	—	—
	Low–middle	2.32	1.58–3.41	<0.001
Policy 3: earlier closing times for bars and nightclubs	High	0.24	0.19–0.29	<0.001
	High–middle	(Ref)	—	—
	Low–middle	1.73	1.09–2.74	0.021
Policy 4: earlier closing times for buying alcohol from bottle stores and supermarkets	High	0.26	0.22–0.30	<0.001
	High–middle	(Ref)	—	—
	Low–middle	1.34	0.87–2.05	0.183
Policy 5: an increase in the price of alcohol	High	0.33	0.27–0.40	<0.001
	High–middle	(Ref)	—	—
	Low–middle	0.99	0.58–1.68	0.963
Policy 6: restrictions on alcohol marketing	High	0.29	0.25–0.33	<0.001
	High–middle	(Ref)	—	—
	Low–middle	0.94	0.61–1.44	0.775
Policy 7: lowering the blood/breath alcohol limit for drink driving	High	1.14	0.97–1.35	0.119
	High–middle	(Ref)	—	—
	Low–middle	2.80	1.95–4.02	<0.001
Policy 8: more random breath testing	High	0.41	0.33–0.50	<0.001
	High–middle	(Ref)	—	—
	Low–middle	2.66	1.80–3.95	<0.001
Policy 9: an increase in alcohol taxes to pay for alcohol treatment	High	0.37	0.31–0.44	<0.001
	High–middle	(Ref)	—	—
	Low–middle	1.14	0.70–1.87	0.591
Policy 10: an increase in alcohol taxes to lower other taxes	High	0.38	0.33–0.44	<0.001
	High–middle	(Ref)	—	—
	Low–middle	1.27	0.76–2.12	0.360
Policy 11: an increase in alcohol taxes to pay for any government purpose	High	0.26	0.22–0.31	<0.001
	High–middle	(Ref)	—	—
	Low–middle	0.84	0.48–1.47	0.537
Policy 12: taxing drinkers to pay for the cost of alcohol related harm to society	High	0.16	0.13–0.20	<0.001
	High–middle	(Ref)	—	—
	Low–middle	0.55	0.29–1.03	0.063

a Weighted data, results not shown for age, gender, education and drinking risk. For Thailand (policy measures 10, 11 and 12) and for Vietnam (policy measure 8) are not included.

AOR, adjusted odds ratio; CI, confidence interval; DV, dependent variable; IV, independent variable.

## Discussion

Support by drinkers in the seven countries was above 50% for 79% of the possible country/policy combinations and it was generally higher for policies addressing drink driving and increasing the alcohol purchase age. Support was, however, lower for policies related to increasing taxes on alcohol, especially where there was no indication where the increased revenue would be spent. These findings are in keeping with research conducted elsewhere in high‐ and middle‐income countries 8, 11, 16, 17, 18, 22. The unique contribution of this study is its comparison of policy support among countries differing in income level using comparable methodology. We found a trend in policy support, generally being highest in the low–middle‐income countries, followed by the high–middle‐income countries and then lowest in the high‐income countries.

It has been recognised that policy support is not static and may change over time 12, 13, 14, and it is possible that the two high‐income counties are at a different place in the policy development process, that is, alcohol policy reform may have had a longer history in New Zealand than in St Kitts and Nevis. Also, they may have had stronger restrictions than the middle‐income countries and there might be push back against further tightening of policies. Policy support in South Africa, a high–middle‐income country, was not always greater than in New Zealand. This could be because other research has shown that there are very high levels of heavy drinking in South Africa 26 and it is well recognised that heavier drinkers are less likely to support more restrictive alcohol policies 8, 10, 11, 12, 14, 15, 16, 17.

The findings of this study were generally in line with the findings of previously conducted research in middle and high‐income countries which, in contrast to our study often also included non‐drinkers, in that there was greater support for more restrictive alcohol policies among older persons 9, 15, 17, 19 and women 9, 12, 15, 16, 17, 19. In contrast to other studies 8, 11, 14, 17 we did not find much variation in support for the various policy proposals in terms of educational level attained. Our findings were, however, consistent with the findings of prior research that found lower levels of support for certain alcohol policies among drinkers who drank more frequently or at high volumes 9, 12, 15, 16, 17, 18, 21.

This study is subject to various limitations. Firstly, in some countries cities, sub‐districts or municipalities were sampled and as a result the finding in these countries might not necessarily generalise to the whole country, and it is furthermore likely that many of the samples were biased towards urban populations which tend to have more liberal views. It should be further recognised that response rates were lower in some countries. The different methodology used in some countries (phone vs. face‐to‐face surveys) may have biased findings towards under reporting in the face‐to‐face interviews. The high reporting of heavy drinking in South Africa suggest that this was not the case.

Some might consider it to be a limitation not to have included non‐drinkers in the study. However, given the finding from previous research that non‐drinkers are likely to be more supportive of alcohol policy controls 8, 10, 11, 12, 14, 15, 16, 17 and the finding that there are likely to be more non‐drinkers in low‐ and middle‐income countries than in high‐income countries 3, the differences between middle‐ and high‐income countries we observed would have been magnified had non‐drinkers been included in each of the country samples. It should also be noted that St Kitts and Nevis is atypical of high‐income countries, being a very small island nation with a small population and which is highly dependent on tourism and banking. Furthermore, it only became listed as a high‐income country by the World Bank in 2014.

Building on the core findings of this study that policy support seems to differ by the income level of country, with policy support being higher in less affluent societies, there is a need to replicate this research with a broader range of countries, including low‐income countries and high‐income countries and to increase the number of countries in each income group. Recognising that policy support changes over time future research should also include an assessment of where countries are at in their own policy development trajectory as well as indicators of the level of perceived harm to others associated with alcohol use.

## Conclusion

This study has shown that it is possible to conduct alcohol policy research using similar methodologies among countries varying in income level, language, education and drinking behaviour. The findings demonstrate substantial support by drinkers for increasing controls on alcohol in a number of areas. As such, they could give impetus to policy makers to move forward on strengthening alcohol control policies. We found a trend in policy support, generally being the highest in the low–middle‐income countries, followed by the high–middle‐income countries and then lowest in the high‐income countries. Further research is needed to replicate and expand on these findings.

## Conflict of interest

The authors have no conflicts of interest.

## References

[1] GBD 2015 Risk Factors Collaborators . Global, regional, and national comparative risk assessment of 79 behavioural, environmental and occupational, and metabolic risks or clusters of risks, 1990–2015: a systematic analysis for the global burden of disease study 2015. Lancet 2016;388:1659–724. 27733284 10.1016/S0140-6736(16)31679-8 PMC5388856

[2] Rehm J , Gmel GE , Gmel G *et al* The relationship between different dimensions of alcohol use and burden of disease‐an update. Addiction 2017;112:968–1001. 28220587 10.1111/add.13757 PMC5434904

[3] World Health Organization . Global status report on alcohol and health 2014. Geneva: World Health Organization, 2014.

[4] World Health Organization . Global strategy to reduce the harmful use of alcohol. Geneva: World Health Organization, 2010. 10.2471/BLT.19.241737 PMC7047030 32132758

[5] Giesbrecht N , Österberg E . WHO's global strategy to reduce the harmful use of alcohol: an assessment of recent policies and interventions in Finland and Ontario, Canada. Nordisk Alkohol Nark 2013;30:297–316.

[6] Parry CDH . Testing the National Alcohol Policy Score Card (NAPSC) to assess progress in implementing a comprehensive policy response to reduce the harmful use of alcohol in South Africa. Int J Alcohol Drug Res 2013;3:202–9.

[7] Parry CDH . Alcohol policy in South Africa: a review of policy development processes between 1994 and 2009. Addiction 2010;105:1340–5. 20653617 10.1111/j.1360-0443.2010.03003.x

[8] Diepeveen S , Ling T , Suhrcke M , Roland M , Marteau TM . Public acceptability of government intervention to change health‐related behaviours: a systematic review and narrative synthesis. BMC Public Health 2013;13:756. 23947336 10.1186/1471-2458-13-756 PMC3765153

[9] Giesbrecht N , Livingston N . Public perceptions and alcohol policies: six case studies that examine trends and interactions. Drug Alcohol Rev 2014;33:217–9. 24761756 10.1111/dar.12139

[10] Ialomiteanu AR , Giesbrecht N , Adlaf EM , Wettlaufer A . Trend in public opinion on alcohol issues during a period of increasing access to alcohol: Ontario, Canada, 1996‐2011. Drug Alcohol Rev 2014;33:249–58. 24761757 10.1111/dar.12130

[11] Callinan S , Room R , Livingston M . Changes in Australian attitudes to alcohol policy: 1995–2010. Drug Alcohol Rev 2014;33:227–34. 24372933 10.1111/dar.12106

[12] Rossow I , Storvoll EE . Long‐term trends in alcohol policy attitudes in Norway. Drug Alcohol Rev 2014;33:220–6. 24329767 10.1111/dar.12098

[13] Greenfield T , Karriker‐Jaffe K , Giesbrecht N , Kerr WC , Ye Y , Bond J . Second‐hand drinking may increase support for alcohol policies: new results from the 2010 National Alcohol Survey. Drug Alcohol Rev 2014;33:259–67. 24761758 10.1111/dar.12131 PMC4024451

[14] Buykx P , Gilligan C , Ward B , Kippen R , Chapman K . Public support for alcohol policies associated with knowledge of cancer risk. Int J Drug Policy 2015;26:371–9. 25217801 10.1016/j.drugpo.2014.08.006

[15] Harwood EM , Bernat DH , Lenk KM , Vazquez MJ , Wagenaar AC . Public opinion in Puerto Rico on alcohol control policies. Hisp J Behav Sci 2004;26:426–45.

[16] Pinsky I , Sanches M , Zaleski M , Laranjeira R , Caetano R . Opinions about alcohol control policies among Brazilians: the first national alcohol survey. Contemp Drug Probl 2007;34:635–48.

[17] Ferrell B . Alcohol policy and regulation: public opinion amongst young adults in Khayalitsha, South Africa (MPH dissertation). Rondebosch: University of Cape Town, 2016 https://open.uct.ac.za/handle/11427/20856.

[18] Tobin C , Moodie AR , Livingstone CA . Review of public opinion towards alcohol controls in Australia. BMC Public Health 2011;11:58. 21272368 10.1186/1471-2458-11-58 PMC3048532

[19] Osterberg E , Lindeman MF , Karlsson T . Changes in alcohol policies and public opinions in Finland 2003‐2013. Drug Alcohol Rev 2014;33:242–8. 24628708 10.1111/dar.12128

[20] Lund IO , Halkjelsvik T , Storvoll EE . Overlap in attitudes to policy measures on alcohol, tobacco and illegal drugs. Int J Drug Policy 2016;28:60–6. 26440774 10.1016/j.drugpo.2015.09.002

[21] Seo S , Chun S , Newell M , Yun M . Korean public opinion on alcohol control policy: a cross‐sectional international alcohol control study. Health Policy 2015;119:33–43. 25442376 10.1016/j.healthpol.2014.10.016

[22] Hope A . The ebb and flow of attitudes and policies on alcohol in Ireland 2002‐2010. Drug Alcohol Rev 2014;33:235–41. 24628739 10.1111/dar.12129

[23] Casswell S , Meier PF , MacKintosh AM *et al* The international alcohol control (IAC) study‐evaluating the impact of alcohol policies. Alcohol Clin Exp Res 2012;36:1462–7. 22404733 10.1111/j.1530-0277.2012.01738.x

[24] Kaminska O , Lynn S . Survey‐based cross‐country comparisons where countries vary in sample design: issues and solutions. J Off Stat 2017;33:123–36.

[25] StataCorp . Stata Statistical Software: Release 14. College Station: StataCorp LP, 2015.

[26] Parry CDH , Trangenstein P , Lombard C , Jernigan D , Morojele N . Support for alcohol policies from drinkers in the City of Tshwane, South Africa: Data from the International Alcohol Control Study. Drug Alcohol Rev 2018;27:S19–S32. 10.1111/dar.12554 PMC5969057 28493419

